# Context-Aware Enhanced Feature Refinement for small object detection with Deformable DETR

**DOI:** 10.3389/fnbot.2025.1588565

**Published:** 2025-06-10

**Authors:** Donghao Shi, Cunbin Zhao, Jianwen Shao, Minjie Feng, Lei Luo, Bing Ouyang, Jiamin Huang

**Affiliations:** ^1^Zhejiang Key Laboratory of Digital Precision Measurement Technology Research, Hangzhou, China; ^2^Advanced Manufacturing Metrology Research Center, Zhejiang Institute of Quality Sciences, Hangzhou, China; ^3^Key Laboratory of Acoustics and Vibration Applied Measuring Technology, State Administration for Market Regulation, Hangzhou, China

**Keywords:** small object detection, Deformable DETR, mask attention, autonomouts driving, feature extraction

## Abstract

Small object detection is a critical task in applications like autonomous driving and ship black smoke detection. While Deformable DETR has advanced small object detection, it faces limitations due to its reliance on CNNs for feature extraction, which restricts global context understanding and results in suboptimal feature representation. Additionally, it struggles with detecting small objects that occupy only a few pixels due to significant size disparities. To overcome these challenges, we propose the Context-Aware Enhanced Feature Refinement Deformable DETR, an improved Deformable DETR network. Our approach introduces Mask Attention in the backbone to improve feature extraction while effectively suppressing irrelevant background information. Furthermore, we propose a Context-Aware Enhanced Feature Refinement Encoder to address the issue of small objects with limited pixel representation. Experimental results demonstrate that our method outperforms the baseline, achieving a 2.1% improvement in mAP.

## 1 Introduction

With the continuous advancement of technology, object detection has become a critical research area (Tong and Wu, 2022; Wei et al., 2024; Zhao Y. et al., 2024), achieving significant progress in practical applications such as autonomous driving (Bogdoll et al., 2022; Song and Lee, 2023; Su et al., 2022), ship black smoke detection (Dimitropoulos et al., 2016; Wang et al., 2023), and drone aerial photography (Li et al., 2024; Tang et al., 2024; Zhu P. F. et al., 2021). However, as the demands of these applications continue to increase, the challenges faced by object detection tasks have grown more complex, particularly in the case of small object detection (Guo et al., 2024; Li et al., 2024; Tang et al., 2024; Tong and Wu, 2022). Small objects, due to their size and frequent occlusion, pose a considerable challenge, significantly affecting detection accuracy and thus warranting further investigation.

The challenges of small object detection primarily stem from several factors. First, small objects often have low contrast and limited distinguishing features, making it difficult to differentiate them from the background. Additionally, these objects are frequently found in complex and cluttered environments, further complicating the task of separating them from surrounding noise. Finally, even minor errors in bounding box localization can result in the incomplete enclosure of the object, which negatively impacts detection accuracy (Hou et al., 2023).

To address this issue, numerous researchers have explored solutions in this field. Zhu X. K. et al. (2021) tackled the challenges posed by significant object scale variations and the presence of dense and small objects in drone images. They replaced the original prediction head in YOLOv5 with Transformer Prediction Heads (TPH), significantly enhancing the model’s performance. Hou et al. (2023) focused on small object detection, proposing an Ensemble Fusion approach that integrates Cascade R-CNN (Cai and Vasconcelos, 2018) and CenterNet (Zhou et al., 2019). This approach achieved state-of-the-art results on the SOD4SB (Kondo et al., 2023) dataset. Gong et al. (2021) addressed the problem of information flow between deep and shallow layers in traditional Feature Pyramid Network (Lin et al., 2017) structures, introducing the novel concept of “fusion features” to control the information transfer from deep to shallow layers. Experimental results validated the effectiveness of their model.

The aforementioned studies made significant contributions to small object detection by improving classical network architectures to address challenges such as scale variations and dense scenes. However, despite their success, all of these approaches rely on CNN frameworks. Due to the limited receptive field, CNNs capture only local information in shallow layers, which hinders their ability to efficiently capture global context, resulting in a lack of overall image perception and macro-level understanding (Lv et al., 2024; Wang S. et al., 2024; Zhao H. et al., 2024).

To address these limitations, an end-to-end Transformer-based detector known as DETR (Carion et al., 2020) has been introduced. It is recognized for its simplified architecture and the removal of hand-crafted components, making it especially effective for tasks where traditional methods struggle (Lv et al., 2024; Wang S. et al., 2024; Zhao H. et al., 2024). Additionally, transformers, with their enhanced ability to model contextual relationships, have demonstrated clear advantages in small object detection (Zhu X. et al., 2021). DAB-DETR (Liu et al., 2022) and DN-DETR (Li et al., 2022) further improves performance by incorporating an iterative refinement scheme and denoising training. Zhu X. et al. (2021) proposed Deformable DETR, in which attention modules focus only on a small set of key sampling points around a reference, resulting in better performance than DETR, particularly for small objects. Therefore, in this paper, we use Deformable DETR as the baseline to enhance the performance of small object detection.

Despite the progress made by Deformable DETR in small object detection, it overlooks key issues arising from the exclusive use of CNNs in the backbone for feature extraction, which fails to adequately capture global information, resulting in suboptimal feature representation. Additionally, it does not fully address the challenge posed by small objects, which may occupy only a few pixels in the image due to significant size disparities. To overcome these limitations, this paper introduces a Mask Attention mechanism (Fan et al., 2021) to enhance the backbone’s feature extraction capability, improving its ability to capture global context while filtering out irrelevant image information. Moreover, recognizing the challenge posed by small objects with limited pixel occupation, we further propose a Context-Aware Enhanced Feature Refinement Encoder (CAEFRE) to further improve small object detection performance. Experimental results demonstrate the effectiveness of the proposed approach, particularly in enhancing the detection of small objects. To summarize, our contributions are three-fold.

(1) To tackle the issue of incomplete global information extraction caused by the exclusive use of CNNs in the backbone, we introduce Mask Attention. This approach not only enhances the module’s information extraction capability but also effectively filters out irrelevant information through the use of the masking mechanism.
(2) To address the issue that Deformable DETR does not account for small objects occupying only a few pixels, we propose the Context-Aware Enhanced Feature Refinement Encoder to mitigate this limitation.
(3) Experimental results demonstrate the effectiveness of the proposed method, with improvements of 2.1% in both the mAP and small object mAP evaluation metrics.

## 2 Related work

### 2.1 CNN-based object detection methods

Over the past several years, CNN-based frameworks have consistently demonstrated remarkable performance in object detection tasks. These algorithms are broadly categorized into two main types: one-stage and two-stage detectors. In 2014, Girshick et al. (2014) pioneered the two-stage detection paradigm by introducing R-CNN, which achieved impressive results on the VOC dataset. Building upon this foundation, Fast R-CNN (Girshick, 2015) and Faster R-CNN (Ren et al., 2015) further refined the framework by optimizing region proposal generation and classification, significantly improving both efficiency and accuracy. However, despite their superior precision, two-stage detectors are hindered by complex architectures and long inference times, limiting their practicality for real-time applications.

In contrast, one-stage detectors directly predict object bounding boxes and class labels from input images, bypassing the need for separate region proposal steps. This streamlined approach significantly enhances inference speed and real-time performance, making such models increasingly favored by researchers. Liu et al. (2016) introduced SSD, which employed a multi-reference detection strategy to improve accuracy across objects of varying scales. Later, Google introduced EfficientDet (Tan et al., 2020), a powerful object detection framework built upon EfficientNet as its backbone. EfficientDet incorporated several key innovations, including the Bi-Directional Feature Pyramid Network (Bi-FPN), which enhanced multi-scale feature fusion, thereby improving detection accuracy while maintaining computational efficiency. Meanwhile, the YOLO series (Bochkovskiy et al., 2020; Ge et al., 2021; Wang A. et al., 2024) has emerged as one of the most influential CNN-based architectures, continuously evolving through iterative improvements to strike an optimal balance between detection speed and accuracy, making it a popular choice for research and practical applications.

Despite these significant advancements, single-stage CNN-based frameworks still face inherent challenges. One of their key limitations is their reliance on Non-Maximum Suppression (NMS) as a post-processing technique to filter out redundant and irrelevant bounding boxes, which can impact detection efficiency. Additionally, CNN-based architectures, due to their hierarchical nature, often struggle to capture global contextual information, leading to suboptimal performance in detecting small objects.

### 2.2 Advantages and advancements of DETRs in small object detection

Attention mechanisms (Vaswani et al., 2017) have been widely adopted in computer vision tasks due to their ability to capture global information from input data, which significantly improves performance in small object detection compared to traditional convolutional networks. Carion et al. (2020) first introduced DETR, an end-to-end detector based on Transformer, which has gained considerable attention because of its unique features. Notably, DETR eliminates the need for handcrafted anchors and NMS, which is believed to enhance the speed of object detection. This innovation has spurred considerable research in the field. DAB-DETR (Liu et al., 2022) formulates DETR queries as dynamic anchor boxes (DAB), bridging the gap between traditional anchor-based detectors and DETR-like models. Group DETR (Chen et al., 2023) introduces multiple object queries, maintaining the end-to-end inference advantage of DETR while also leveraging the benefits of single-to-multiple queries during training, improving performance and accelerating model convergence.

Deformable DETR (Zhu X. et al., 2021), on the other hand, proposes a deformable attention module, which can naturally aggregate multi-scale features to process feature maps and has demonstrated strong performance, particularly in small object detection. Numerous subsequent studies have focused on improving Deformable DETR, yielding impressive results (Caron et al., 2021; Oquab et al., 2023). However, despite its superior performance in small object detection, Deformable DETR still faces limitations. Its backbone only uses convolutional layers, which results in a loss of global feature information. Moreover, it fails to fully address challenges specific to small object detection, such as small pixel occupation and occlusion, making it necessary to refine these aspects for further enhancement in small object detection tasks.

## 3 Methodology

We first introduce the architecture of DETR, then we explain the overall architecture of the proposed network framework, Context-Aware Enhanced Feature Refinement Deformable DETR (CAEFR-DETR), which extends Deformable DETR by incorporating a Mask Attention mechanism and a Context-Aware Enhanced Feature Refinement Encoder (CAEFRE). Then, we provide a detailed explanation of the Mask Attention added to the backbone, as well as the design of the CAEFRE module.

### 3.1 The architecture of DETR

The DETR (DEtection TRansformers) system employs a Transformer-based framework that incorporates both encoder-decoder components and a bipartite matching mechanism via the Hungarian loss function to ensure individual predictions for all ground-truth bounding boxes. The architecture’s primary features are detailed below.

A standard Transformer encoder-decoder framework processes feature maps $x\in {\mathbb{R}}^{C\times H\times W}$ generated by a CNN backbone, transforming them into object query features. The output features from the decoder undergo both a three-layer feed-forward network (FFN) and linear projection. The regression branch, implemented through the FFN, outputs bounding box coordinates ($b\in {\left[0,1\right]}^{4}$), comprising center coordinates, width, and height values. Meanwhile, the classification branch, realized through linear projection, generates class predictions.

The feature maps, containing pixel-based queries and keys, originate from a ResNet backbone enhanced with positional embeddings. The Transformer decoder receives dual inputs: feature maps generated by the encoder and N learnable object queries (typically 100). The module contains two main attention mechanisms: cross-attention and self-attention. Object queries within the cross-attention module extract relevant information by treating the feature maps as keys. The self-attention mechanism enables object queries to engage in mutual interaction, identifying their relationships. With both queries and keys being object queries themselves, this structure remains feasible for handling a limited number of object queries.

### 3.2 Overview of the proposed method

The overall architecture, as illustrated in Supplementary Figure 1, comprises three primary components: a backbone incorporating Mask Attention and ResNet for feature extraction, a CAEFRE module consisting of three parallel branches designed to enhance small object detection capabilities, and a deformable transformer decoder that integrates both cross-attention and self-attention modules.

The input image’s features are initially extracted by the backbone, which integrates a six-layer ResNet network with the Mask Attention mechanism. The ResNet block is modified by incorporating the Mask Attention mechanism, where each block first applies convolutional neural network (CNN) modules, followed by the Mask Attention mechanism to filter out irrelevant information, and then another CNN layer. This sequence enhances the representation of pertinent features by selectively focusing on relevant information. The refined feature maps are then passed into a Neck module, implemented as a Channel Mapper that consists of CNN layers to further map and refine the feature representations.

After the features are processed, they undergo positional encoding and flattening, after which they are passed to the CAEFRE module for further refinement. The architecture of the CAEFRE module is depicted in Supplementary Figure 4. This module consists of three branches, each performing distinct operations to refine the feature representations: The first branch applies a 1 × 1 convolution to extract fine-grained, local features. The second branch includes a module that sequentially combines two 3 × 3 convolutions and three 1 × 1 convolutions to enhance the scale information of the feature maps, thereby improving the model’s ability to capture medium-scale features. The features extracted from these two branches are then fused and further processed using a multi-head attention mechanism for feature aggregation and refinement. The third branch processes the multi-scale features through six Deformable DETR modules, enabling the extraction of richer spatial and semantic information. The outputs from all three branches are then aggregated to form a comprehensive feature sequence.

Finally, the generated image feature sequence is passed to the decoder, where it is used to form object queries, culminating in the final object detection task.

### 3.3 Backbone with integrated mask attention

To enhance the backbone’s ability to extract global image features, we integrate Mask Attention as a key component. This not only improves the backbone’s capability to capture global information but also utilizes the masking mechanism to filter out unnecessary pixel data.

We use ResNet50 as the base backbone architecture, making modifications to its block. As shown in Supplementary Figure 2, this is the basic block modified by us. After the input data undergoes convolution, it is fed into the Mask Attention module as three separate input sources. The three inputs are processed by three distinct linear transformations to produce the Query(Q), Key(K), and Value(V) matrices. Subsequently, the attention weights are computed by performing the dot product between the Q and V matrices, followed by scaling the result by the square root of the dimension of the key vector, denoted as $d_{K}$. The formula for this computation is as follows:


$$A_{ij}=\frac{Q_{i}\cdot k_{j}^{T}}{\sqrt{d_{K}}}$$

where $A_{ij}$ represents the attention score between the $i_{th}$ query and the $j_{th}$ key.

Subsequently, a masking mechanism is applied to further refine the attention scores. This mask M selectively filters out irrelevant information by modifying the attention matrix as follows:


$$A_{ij}^{mask}=A_{ij}\cdot M_{ij}$$

Here, $M_{ij}$ is a binary mask value: if $M_{ij}$=0, the corresponding attention weight is ignored.

Finally, the attention-weighted sum of the V vectors is computed to generate the output feature. The weighted sum is calculated using the masked attention weights:


$$Y_{j}=\underset{j}{\sum }A_{ij}^{mask}\cdot V_{j}$$

In this equation, $Y_{j}$ is the output corresponding to the query, and $V_{j}$ represents the value associated with the $j_{th}$ position.

The results obtained from the previous computation are then processed through a convolution operation using CNN layers, followed by the application of the ReLU function, generating multi-scale image feature maps.

As illustrated in Supplementary Figure 3, the schematic of the Mask Attention module shows that after extracting the input feature data, Mask Attention selectively enhances the image features. When the input image features do not contain the target objects to be detected, the masking mechanism removes the irrelevant data, effectively refining the information extraction and reduction process from the input image.

### 3.4 Context-aware enhanced feature refinement encoder

To improve the performance of the Deformable DETR network in small object detection, it is crucial to address the challenges posed by small object sizes and scale variations within images. Our approach combines the deformable attention mechanism with convolutional layers. On one hand, small-scale convolutions are used to extract features from small pixel-sized objects, while on the other hand, convolutional layers, being more sensitive to texture information, help capture texture features in the input image. Furthermore, the deformable attention mechanism ensures the preservation of features across various object scales, thereby enhancing the stability of the model’s performance.

As illustrated in Supplementary Figure 4, the overall architecture of the designed CAEFRE is shown. Image features extracted by the backbone and neck modules pass through three information extraction branches.

In the first branch, the input x is processed through a combination of a 1 × 1 convolution to capture fine-grained features from small objects, yielding the feature map $F_{small}$:


$$F_{small}=Conv_{1\times 1}\left(x\right)$$

In the second branch, is processed through a sequential combination of two 3 × 3 convolution followed by three 1 × 1 convolution, generating the feature map $F_{medium}$:


$$F_{medium}=\left(Conv_{3\times 3}\left(Conv_{3\times 3}\left(x\right)\right)\right)*\left(Conv_{1\times 1}\left(Conv_{1\times 1}\left(Conv_{1\times 1}\left(x\right)\right)\right)\right)$$

Where ∗ denotes the successive application of these convolutional layers. The feature maps $F_{small}$ and $F_{medium}$ are then combined via element-wise addition:


$$F_{combined}=F_{small}+F_{medium}$$

This combined feature map is subsequently fed into a multi-head attention mechanism, which facilitates the modeling of relationships across different regions within the image:


$$F_{att}=MultiHeadAttention\left(F_{combined}\right)$$

In parallel, the input x is processed through six Deformable DETR blocks, which effectively accounts for the spatial variations of objects within the image. The output of this block is the feature map $F_{detr}$:


$$F_{detr}=DeformableDetr\left(x\right)$$

Finally, the outputs from the multi-head attention mechanism and the Deformable DETR block are aggregated through element-wise addition:


$$F_{final}=F_{att}+F_{detr}$$

The final feature map is then passed into the decoder for further processing.

## 4 Experiment validation and result analysis

### 4.1 Experimental data and metrics

We use a custom dataset, referred to as the Road Scene Dataset, which we created by capturing road scenes with a dashcam. This dataset includes a diverse range of environments, such as standard roads, highways, urban streets, and rural areas. As shown in Supplementary Figure 5 the collected dataset is presented. The dataset also incorporates common elements of daily life, such as trees, pedestrians, buildings, and the sky. Furthermore, it contains numerous occluded and small-scale objects, adding complexity to the detection tasks. In total, the dataset comprises 6,480 images, which are randomly split into training, testing, and validation sets in an 8:1:1 ratio. Meanwhile, the dataset comprises a total of 69,994 annotated objects across 17 different object categories, reflecting the varied nature of road scenes.

To quantitatively assess the proposed framework, this study employs the mean average precision (mAP) across all classes as the evaluation metric. In addition, mAP values at specific intersection ratio (IoU) thresholds, namely mAP_50_ and mAP_75_, are also introduced to provide a more detailed evaluation. The mAP_50_ represents the mean average precision at an IoU threshold of 50%, reflecting the model’s overall detection capability. Similarly, the mAP_75_ represents the mean average precision at a higher IoU threshold of 75%, offering insights into the model’s precision under stricter localization requirements. Furthermore, to analyze the detection performance of the framework for objects of varying sizes, three mAP values are defined as follows:

(1) mAP_l_: mean average precision value of large objects with an area greater than 96 × 96 pixels.
(2) mAP_m_: mean average precision value of medium-sized objects with between 32 × 32 pixels and 96 × 96 pixels.
(3) mAP_s_: mean average precision value of small objects with less than 32 × 32 pixels.

Based on the aforementioned size classifications, the object size distribution in our dataset is as follows: 33.26% of the annotated objects are classified as small, 42.65% as medium-sized, and 24.09% as large. Additionally, we have annotated the occlusion levels of the objects within the images. Specifically, 61.25% of the annotated objects exhibit some degree of occlusion, while 38.75% are fully visible. Among the annotated objects, 18.77% exhibit occlusion in the range of 0–30%, 16.70% fall within the 30–60% occlusion range, and 25.78% have an occlusion range of 60–100%.

The diverse characteristics of the dataset, including varied object sizes, occlusions, and environmental conditions, make it a challenging yet representative dataset for evaluating the generalization ability of our proposed model. These complexities ensure that the model is tested across different real-world scenarios, making it more robust in handling diverse conditions such as cluttered scenes, varying object scales, and partial occlusions.

### 4.2 Experimental setup

The experimental setup for this study is summarized in Supplementary Table 1. The framework was trained using the AdamW optimizer with an initial learning rate of 0.0001. Parameter-wise learning rate adjustment was applied, and input images had a resolution of 1920 × 1,020 pixels with a batch size of 1. The MultiStepLR scheduler was used to manage the learning rate, with a reduction by a factor of 0.1 at the 40th epoch during the 50-epoch training process. This setup ensures efficient training and model convergence.

All models in the experiments were initialized using officially released pre-trained weights. Specifically, DETR, SSD, Deformable DETR, and our proposed method were based on the MMDetection framework (Chen et al., 2019), while the YOLOv8 models used pre-trained weights from the Ultralytics framework (Jocher et al., 2023). Meanwhile, except for our method, all models were trained using their default configurations without any additional modifications.

### 4.3 Ablation experiments

To thoroughly evaluate the effectiveness of each module in CAEFR-DETR, this study conducts ablation experiments by incorporating single and combined modules into the baseline Deformable DETR. The backbone network with Mask Attention and the CAEFRE are individually added to the original Deformable DETR framework and compared against the baseline algorithm. The experimental results are summarized in Supplementary Table 2. Among them, Model 1 represents the original Deformable DETR algorithm, while Model 4 corresponds to the proposed CAEFR-DETR method. The remaining models are comparative algorithms used in the ablation experiments. A checkmark (“√”) in the corresponding position indicates the inclusion of a specific module.

Supplementary Table 2 demonstrates that incorporating the Mask Attention module in Deformable DETR enhances feature extraction and noise suppression capabilities, increasing the detection accuracy to 65.8%. Notably, while the Mask Attention module improves mAP50 and mAP75 by enhancing global contextual focus and reducing background noise, it may slightly reduce mAPs, possibly because the masking mechanism suppresses subtle cues critical for detecting small, low-contrast objects. Similarly, the CAEFRE in Model 3 further improves detection accuracy, raising it to 65.5%. The proposed algorithm effectively addresses challenges related to occlusion and small object detection, resulting in outstanding detection performance. On the dataset, the mean average precision (mAP) improves from 65.3 to 66.7%, reflecting a 1.4% increase. These results validate the effectiveness of the proposed algorithm in enhancing object detection performance.

However, it is important to note that the performance gains from Mask Attention (Model 2) and CAEFRE (Model 3) alone are modest, with increases of 0.5 and 0.2% in mAP, respectively. In real-world object detection tasks, particularly those involving small and occluded objects, these modules may not show significant improvements in performance compared to large, unobstructed objects. Nonetheless, when combined, Mask Attention and CAEFRE in Model 4 lead to a more substantial performance boost of 1.4% (from 65.3 to 66.7% in mAP). This demonstrates that while individual modules may not provide dramatic improvements, their combined effect significantly enhances the model’s ability to address challenges related to occlusion and small object detection.

Furthermore, it is essential to evaluate the computational efficiency of the proposed algorithm. In this study, key metrics such as FLOPs, FPS, and model parameters are considered to evaluate the computational efficiency. The results show that all models maintain consistent computational complexity, with FLOPs at 418G and model parameters at 42.5 M. Notably, the FPS for all models remains steady at 23.3, indicating that, despite the introduction of additional modules, the inference speed is relatively stable and does not experience significant performance degradation. Therefore, while the individual performance gains from Mask Attention and CAEFRE are modest, their integration into the model enhances its robustness and detection performance without incurring substantial computational overhead. This balance between improved detection accuracy and maintained computational efficiency highlights the practical scalability of the proposed CAEFR-DETR method.

Supplementary Figure 6 presents the mAP iteration curves for both the baseline and our proposed model (CAEFR-DETR) over 50 training epochs. As illustrated in the figure, the mAP of the proposed model exhibits a faster growth rate during the early and mid-training stages compared to the baseline. Additionally, the curve of CAEFR-DETR appears smoother, indicating improved training stability and faster convergence. Both models experience a significant boost in mAP after epoch 40, suggesting a critical learning phase. By the end of training, the maximum mAP achieved by CAEFR-DETR reaches 0.667, surpassing the baseline’s 0.653 by 2.1%. This result highlights the enhanced learning capability of our model, leading to superior performance in comparison to the baseline.

### 4.4 Comparison with the performance of other algorithms

To further validate the superiority of the proposed algorithm, this study compares it with several classical algorithms commonly used in the field of object detection. These include SSD as one-stage detectors, YOLOv8 models (N, S, M, L, and X), and Transformer-based end-to-end detectors, DETR and Deformable DETR. In the comparison experiment, mAP, mAP_50_, mAP_75_, mAP_s_, mAP_m_, mAP_l_ are used as the evaluation indicators of each algorithm, and the model training and test are kept unchanged on the same dataset. Supplementary Table 3 shows the detection results.

As shown in Supplementary Table 3, the dataset evaluation results demonstrate that our proposed method outperforms existing approaches across multiple metrics. Specifically, our model achieves a 1.4% higher mAP compared to Deformable DETR, an 11.9% improvement over SSD, and a 2.8% gain relative to YOLOV8-L, which is one of the top-performing YOLO models in this comparison.

Notably, in small object detection, our method exhibits significant improvements, surpassing Deformable DETR by 1.1%, SSD by 23.9%, and YOLOV8-X by 16.8%. This indicates the effectiveness of our approach in enhancing feature extraction and localization for small-scale objects, which are often challenging to detect.

Additionally, our method demonstrates strong performance in medium-scale and large-scale object detection, achieving a competitive mAP_m_ of 0.677 and an mAP_l_ of 0.817. While YOLOV8-L achieves the highest mAP_l_, our model still performs robustly across all object sizes, making it a well-rounded and highly effective detection framework. These experimental results highlight the overall superiority of our approach, particularly in handling small object detection while maintaining strong generalization across different object scales.

To illustrate the effectiveness of the training process, we plotted the training loss curves for various models, as shown in Supplementary Figure 7. Given the differences in loss functions used by different architectures, we adopted classification loss as a common metric for comparison. This provides a clear insight into the learning efficiency and convergence behavior of each model.

The figure reveals several key observations. SSD exhibits the highest loss values throughout training, indicating greater difficulty in optimizing its classification predictions. DETR and YOLOV8-X show relatively stable convergence, but their loss values remain consistently higher than ours. In contrast, our proposed method demonstrates the lowest classification loss, reflecting its superior learning efficiency and more effective feature extraction.

Furthermore, our model exhibits a faster convergence rate, with a steep decline in loss during the initial epochs, suggesting that it rapidly learns discriminative features. Over the later epochs, our method maintains a consistently lower loss compared to other models, highlighting its ability to achieve a more optimized classification process with improved generalization.

These findings reinforce the effectiveness of our approach in object detection, showing that it not only optimizes classification performance more effectively but also achieves more stable and efficient training compared to the other models.

As shown in Supplementary Figure 8, the figure presents a comparison of object detection results for the same scene using different methods. Supplementary Figure 8A represents the ground truth, while Supplementary Figure 8B displays the object detection results obtained using the DETR method. Supplementary Figure 8C illustrates the results from the YOLOV8-X method, and Supplementary Figure 8D shows the results using the method we proposed. The confidence threshold for all methods is set to 0.5. In these images, we have highlighted three small objects with significant differences in predictions across the methods to better showcase the results.

In Supplementary Figure 8B, which presents the DETR-based object detection results for the street scene, a comparison with the ground truth in Supplementary Figure 8A reveals that the bicycle in area *d* is not detected, while the anchor boxes in areas *e* and *f* appear excessively redundant. Supplementary Figure 8C shows the object detection results using the YOLOV8-X method. Compared to Supplementary Figure 8B, YOLOV8-X correctly predicts the object in area *g*, and the anchor boxes in areas *h* and *i* are no longer redundant. However, when compared to the ground truth, it is evident that YOLOV8-X fails to detect the small objects in areas *h* and *i*, resulting in the absence of corresponding anchor boxes.

Supplementary Figure 8D illustrates the object detection results using our proposed method. As shown, our method successfully detects the objects in areas *j*, *k*, and *l*, with no redundant anchor boxes. The experimental results demonstrate that our proposed method effectively improves model performance, offering a significant advantage in detecting small objects.

Beyond mAP, the Recall metric is indispensable for evaluating model performance. Recall, defined as the ratio of correctly identified objects to the total number of actual objects, provides a critical measure of a model’s ability to detect all relevant instances within a given scene. It is especially vital in applications where missing an object could lead to significant consequences. Mathematically, Recall can be expressed as:


$$Recall=\frac{TP}{TP+FN}$$

Where TP denotes true positives (correctly detected objects) and FN represents false negatives (missed objects). In complex environments like road scenes, a high Recall minimizes the risk of missing critical objects, thereby improving the model’s reliability for safety-critical tasks.

To demonstrate the robustness of our method in autonomous driving scenarios, we compare our approach with the baseline by calculating the Recall values for both models. As shown in Supplementary Figure 9, which presents the Confusion Matrix Comparison between Deformable DETR and Our Proposed Method, (A) represents the confusion matrix of Deformable DETR, while (B) represents the confusion matrix produced by our method. The *x*-axis represents the predicted object labels, and the *y*-axis represents the ground truth labels. Our method shows significant improvements in Recall, particularly for small objects.

Moreover, as shown in Supplementary Table 4, our method significantly improves the Recall for small objects. However, the improvements for larger objects, such as buses and trucks, are less pronounced. Our method achieves a Recall of 0.899 for bicycles, improving from 0.887 in the baseline. Additionally, for small trucks, Recall increases from 0.949 to 0.983, further highlighting the advantage of our method in detecting small objects. These improvements are most pronounced in categories where the objects are smaller and more prone to occlusion, underscoring the effectiveness of our approach in addressing the challenges posed by small object detection.

Finally, to illustrate the effectiveness of our model, we present qualitative results showcasing object detection outcomes generated using the proposed approach. The dataset includes 17 common road objects, such as pedestrians, bicycles, SUVs, and cars. Each detected object is categorized, and the final predicted confidence scores for each class are provided. As shown in Supplementary Figure 10, these results visually demonstrate the model’s ability to accurately identify and classify objects in complex road environments.

## 5 Conclusion

In this paper, we propose CAEFR-DETR, an improved Transformer-based object detection model designed to address the challenges of small object detection in complex environments. By integrating Mask Attention into the backbone, our approach enhances global feature extraction while effectively suppressing background noise. Additionally, the Context-Aware Enhanced Feature Refinement Encoder (CAEFR) improves multi-scale feature representation, significantly enhancing the detection of small objects with limited pixel coverage.

Extensive experiments validate the effectiveness of our proposed model. Compared to the baseline, CAEFR-DETR achieves a 2.1% improvement in mAP, demonstrating its superior detection performance. Our method also outperforms existing models, including Deformable DETR, SSD, and YOLOV8 variants, with particularly notable improvements in small object detection, where it surpasses Deformable DETR by 1.1%, SSD by 23.9%, and YOLOV8-X by 16.8% in mAP_s_. Additionally, our model shows significant improvements in Recall, particularly for small objects such as bicycles and small trucks, with Recall values of 0.899 and 0.983, respectively, outperforming Deformable DETR. Furthermore, the loss convergence analysis reveals that CAEFR-DETR exhibits faster and more stable convergence, achieving lower classification loss compared to other models. This underscores its optimization efficiency, improved feature learning, and enhanced robustness in complex detection scenarios.

Overall, our proposed CAEFR-DETR demonstrates excellent performance in small object detection, making it well-suited for real-world applications such as autonomous driving and ship black smoke detection. The improvements in both accuracy and training efficiency highlight its potential for broader deployment in scenarios requiring precise and reliable small object detection in dynamic and cluttered environments. Future work will explore further enhancements in computational efficiency and generalizability across diverse datasets to expand its applicability in real-world tasks.

## Data Availability

Publicly available datasets were analyzed in this study. This data can be found at: send email to 18323135471@163.com to get the data.
