# Supplementary material for: Context-Aware Enhanced Feature Refinement for small object detection with Deformable DETR
Source: Front Neurorobot. 2025 Jun 10;19:1588565. doi: 10.3389/fnbot.2025.1588565 (PMC12185399; doi:10.3389/fnbot.2025.1588565)
Supplement: Supplementary file 1 [file Data_Sheet_1.docx]

Supplementary Material

# Supplementary Figures and Tables

## Supplementary Figures


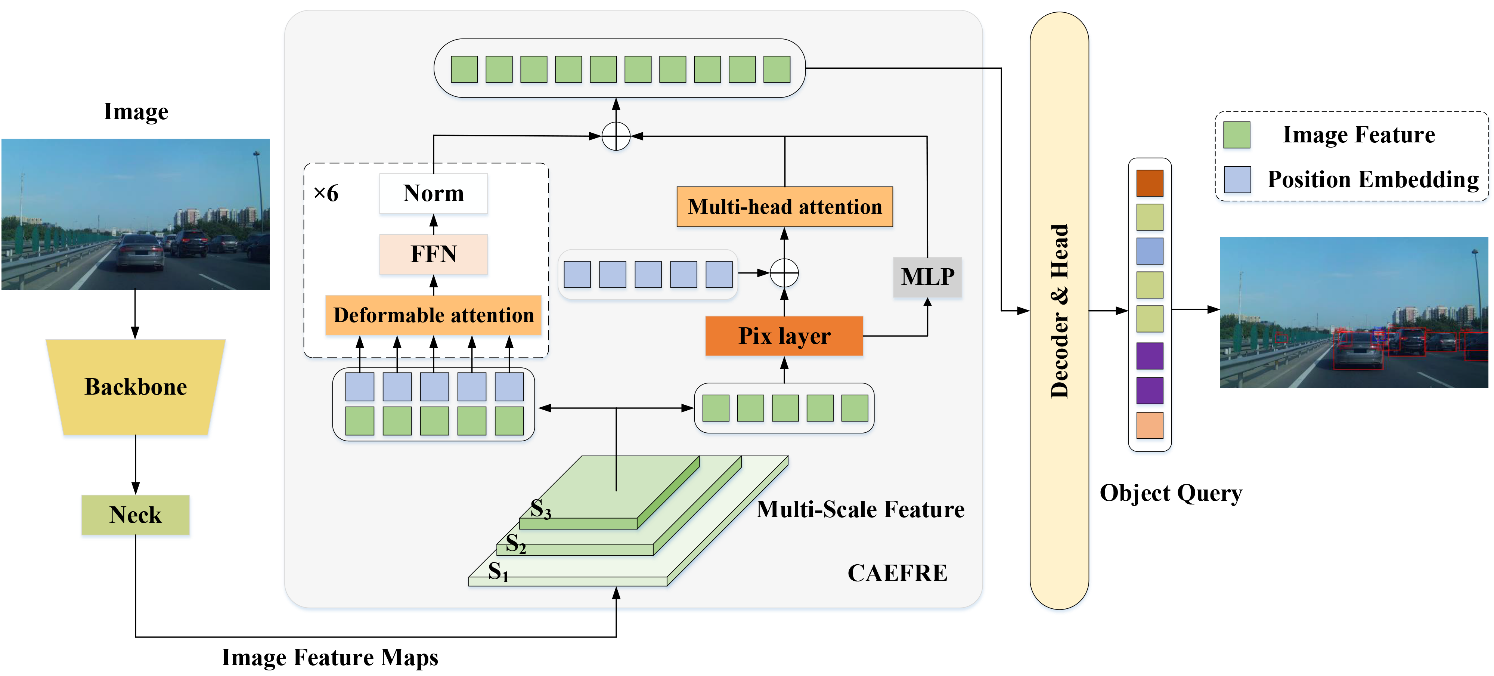


**Supplementary Figure 1.** Overview of the CAEFR-DETR. The architecture includes a backbone integrated with Mask Attention and ResNet, a Context-Aware Enhanced Feature Refinement Encoder (CAEFRE), and a deformable Transformer decoder.


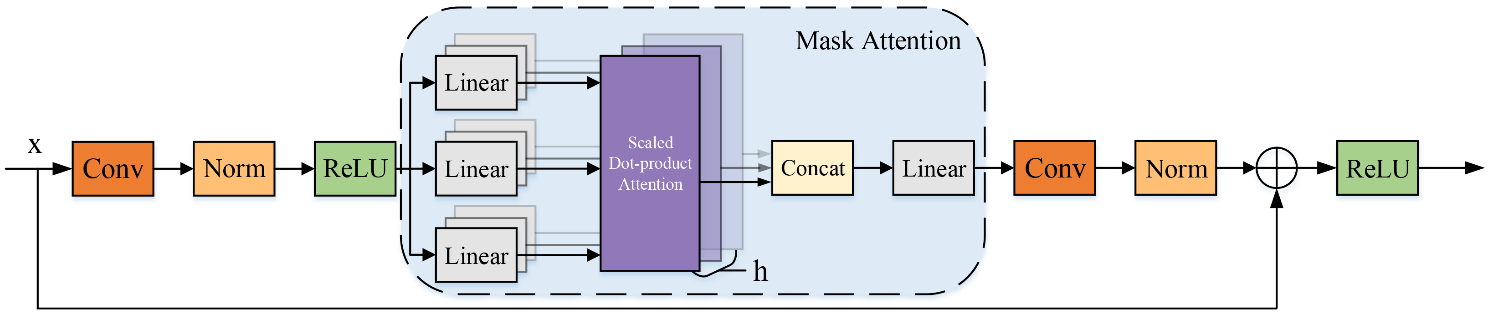


**Supplementary Figure 2.** The basic block incorporating Mask Attention


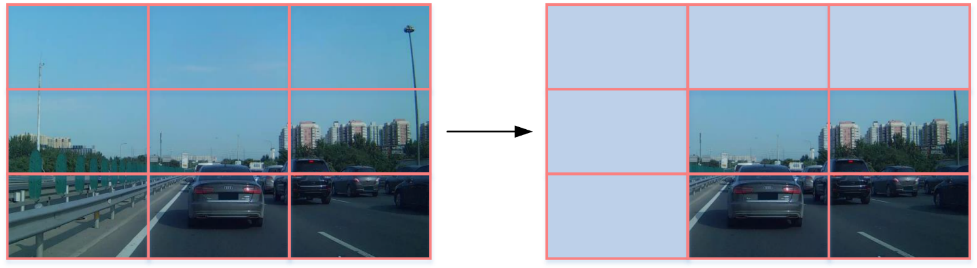


**Supplementary Figure 3.** Principle of Mask Attention in image processing


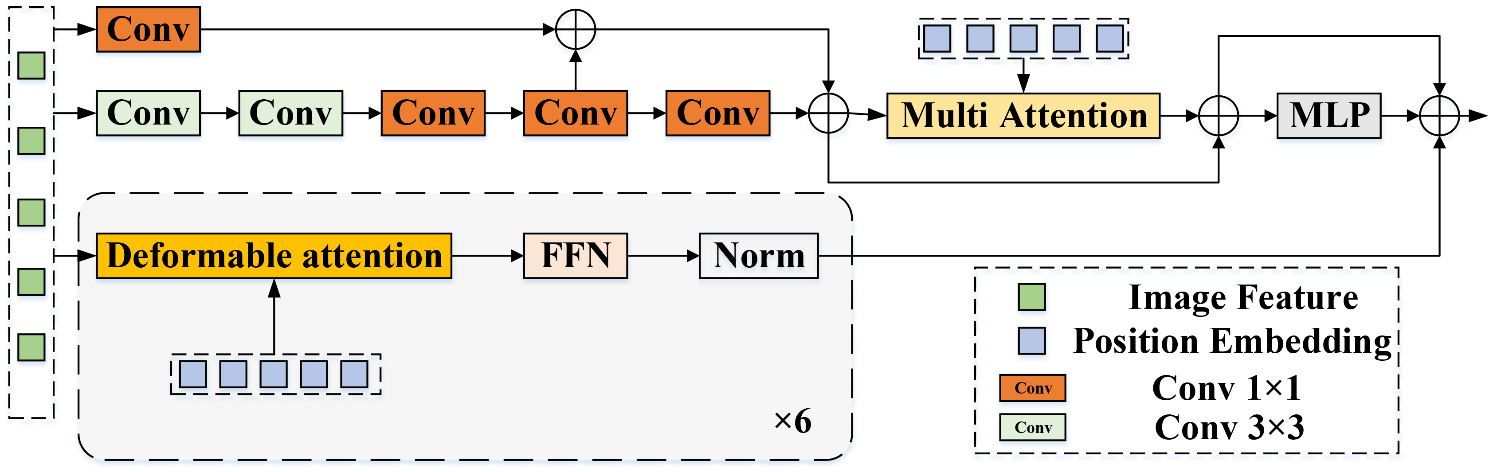


**Supplementary Figure 4.** The overall framework of CAEFRE. It consists of three parallel branches for multi-scale and spatial feature extraction, followed by feature fusion and refinement for small object enhancement.


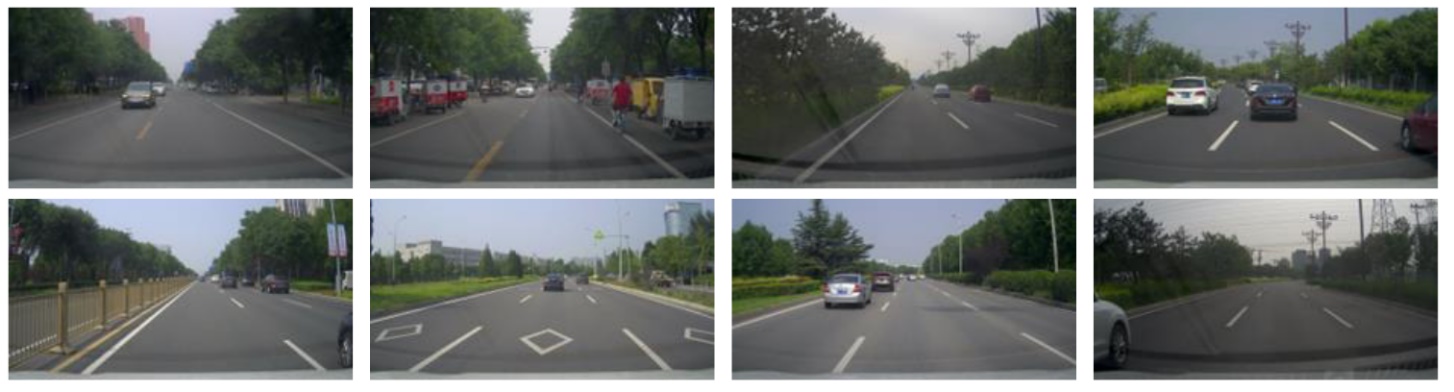


**Supplementary Figure 5.** Original dataset example. Sample images from the custom Road Scene Dataset, including various object types and occlusion conditions. The dataset covers diverse environments such as urban streets, highways, and rural roads


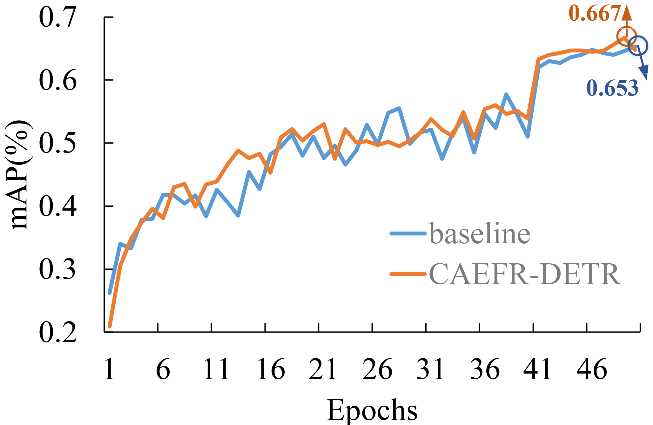


**Supplementary Figure 6.** mAP value iteration curve. The x-axis represents the training epochs (0 to 50), and the y-axis indicates the mAP value (0 to 1). CAEFR-DETR shows faster and smoother convergence than the baseline.


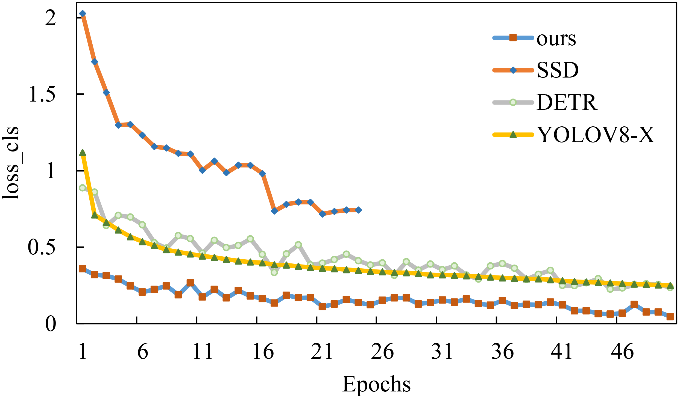


**Supplementary Figure 7.** Training loss curves of various models. The x-axis shows training epochs, and the y-axis denotes classification loss. The proposed CAEFR-DETR achieves lower and more stable loss.


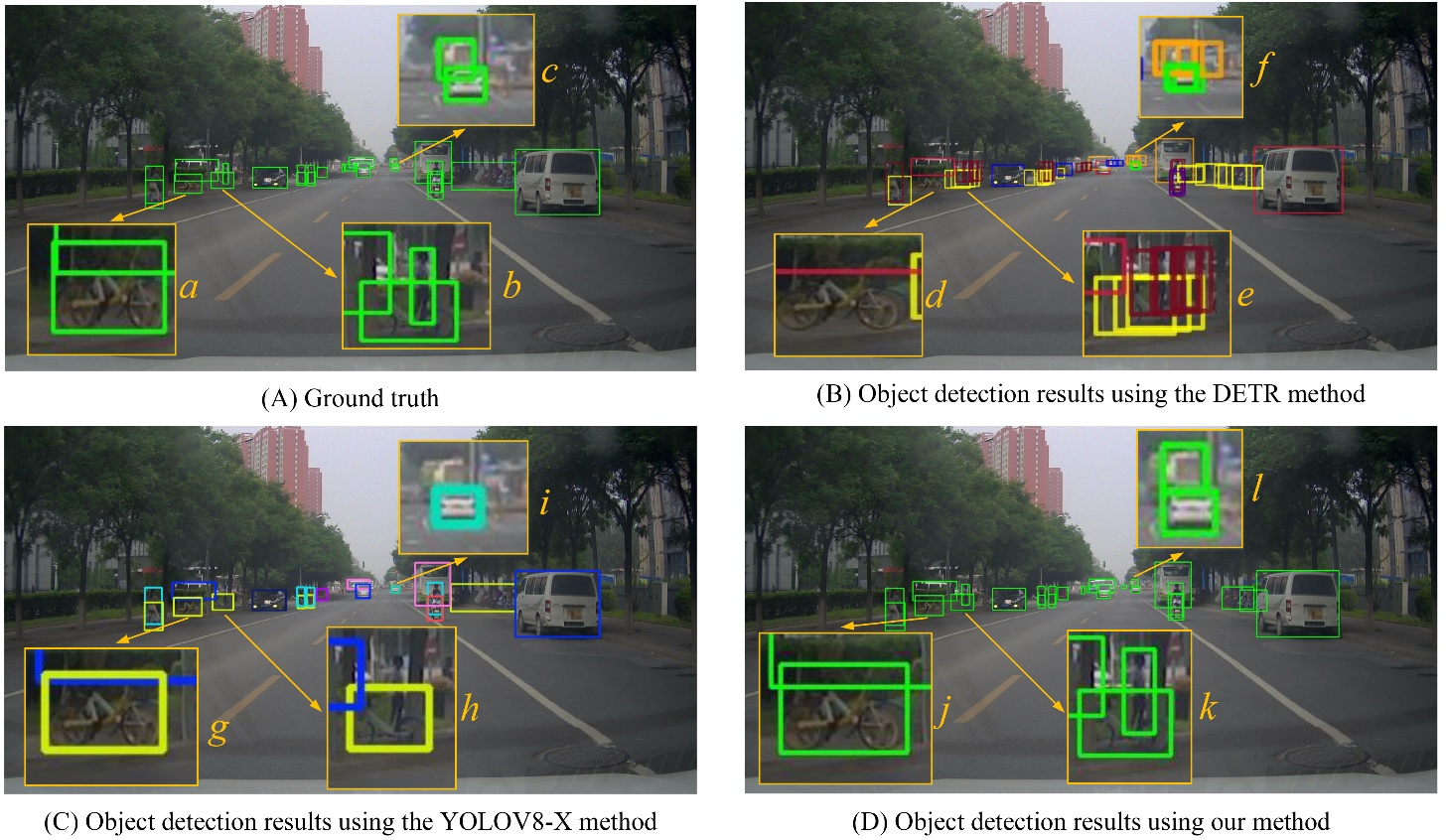


**Supplementary Figure 8.** Comparison chart of object detection results across different methods. Small object areas show better detection with fewer redundant boxes in our method.

**
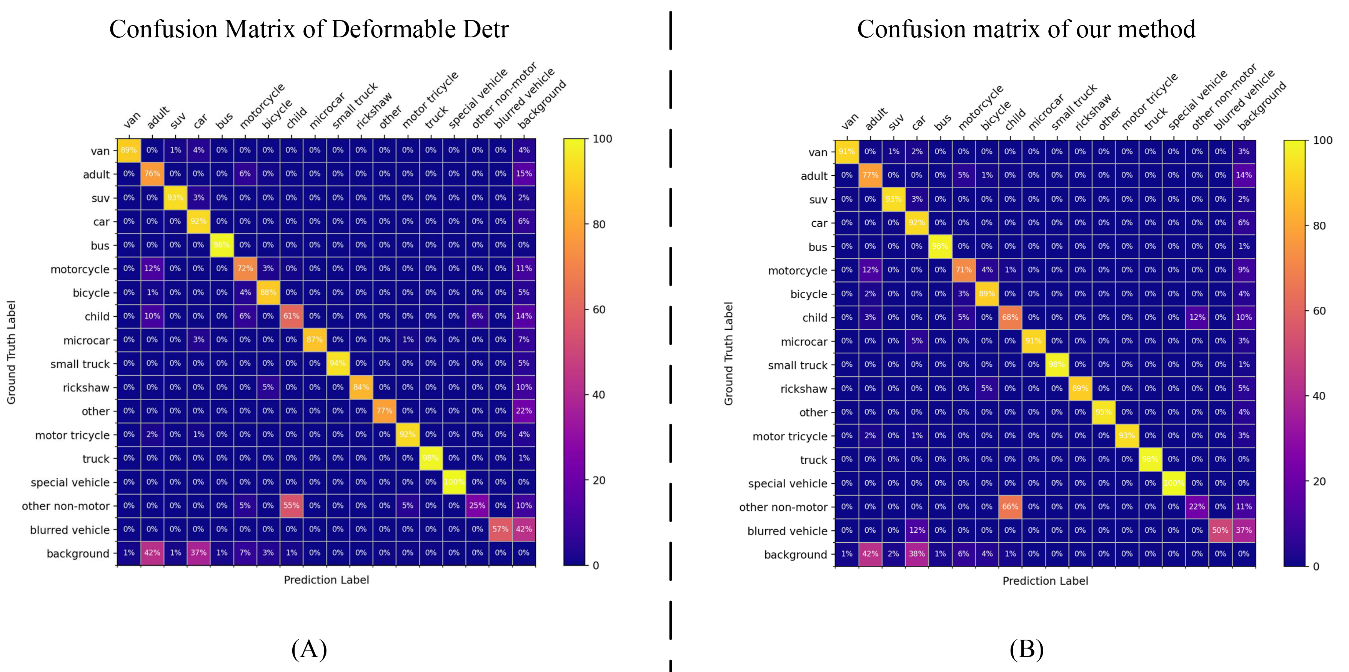
**

**Supplementary Figure 9.** Confusion Matrix Comparison between Deformable DETR and Our Proposed Method. (A) Confusion Matrix of Deformable DETR; (B) Confusion Matrix of Our Proposed Method


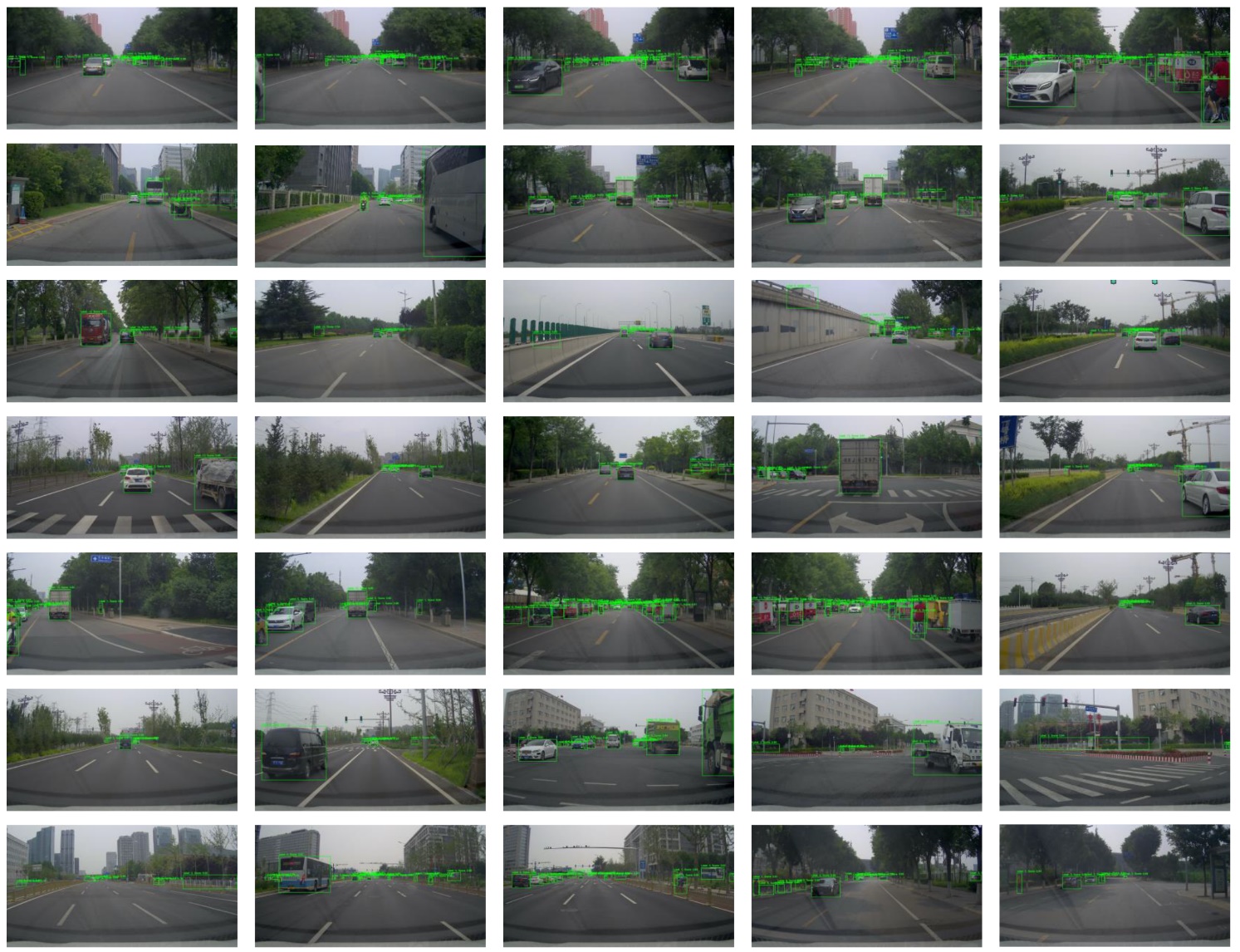


**Supplementary Figure 10.** Object detection outcomes using the proposed method. The figure shows accurate classification and bounding boxes for multiple objects under complex road scenes, validating the robustness of the model.

## Supplementary Tables

**Table 1.** Experimental environment

| **Name** | **Version** |
| --- | --- |
| Operating Systems | Ubantu20.04 |
| GPU | NVIDIA GeForce RTX A6000 |
| Python | 3.9 |
| Pytorch | 1.13.1 |
| CUDA | CUDA11.7 |

**Table 2.** Ablation experiments results

| **Model** | **Mask Attention** | **CAEFRE** | **mAP** | **mAP_50_** | **mAP_75_** | **mAP_s_** | **mAP_m_** | **mAP_l_** | **Inference**  **FPS** | **FLOPs** | **params** |
| --- | --- | --- | --- | --- | --- | --- | --- | --- | --- | --- | --- |
| 1 |  |  | 0.653 | 0.927 | 0.711 | 0.523 | 0.681 | 0.811 | 23.3 | 418G | 42.5M |
| 2 | √ |  | 0.658 | 0.928 | 0.733 | 0.521 | **0.691** | 0.812 | 23.3 | 418G | 42.5M |
| 3 |  | √ | 0.655 | 0.925 | 0.715 | 0.524 | 0.68 | 0.813 | 23.3 | 418G | 42.5M |
| 4 | √ | √ | **0.667** | **0.932** | **0.759** | **0.534** | 0.677 | **0.817** | 23.3 | 418G | 42.5M |

**Table 3.** Comparison with the performance of other algorithms

| **Model** | **Epochs** | **Pre-trained Model** | **mAP** | **mAP_50_** | **mAP_75_** | **mAP_s_** | **mAP_m_** | **mAP_l_** |
| --- | --- | --- | --- | --- | --- | --- | --- | --- |
| DETR | 50 | detr_r50_8xb2_150e_coco | 0.47 | 0.774 | 0.471 | 0.231 | 0.469 | 0.726 |
| Deformable DETR | 50 | deformable_detr_8xb2_150e_coco | 0.653 | 0.927 | 0.711 | 0.523 | 0.681 | 0.811 |
| SSD | 24 | ssd512_coco | 0.548 | 0.832 | 0.575 | 0.295 | 0.612 | 0.751 |
| YOLOV8-N | 50 | yolov8n | 0.534 | 0.694 | 0.592 | 0.176 | 0.599 | 0.851 |
| YOLOV8-S | 50 | yolov8s | 0.577 | 0.743 | 0.631 | 0.244 | 0.664 | 0.873 |
| YOLOV8-M | 50 | yolov8m | 0.611 | 0.782 | 0.662 | 0.31 | 0.688 | 0.887 |
| YOLOV8-L | 50 | yolov8l | 0.639 | 0.792 | 0.704 | 0.342 | 0.703 | **0.9** |
| YOLOV8-X | 50 | yolov8x | 0.636 | 0.795 | 0.685 | 0.366 | **0.716** | 0.894 |
| Ours | 50 | deformable_detr_8xb2_150e_coco | **0.667** | **0.932** | **0.759** | **0.534** | 0.677 | 0.817 |

Notes: The bolded values indicate the best performance. Our proposed model achieves superior results on the evaluation metrics mAP, mAP_50_, mAP_75_, and mAP_s_. YOLOV8-X achieves the highest score for mAP_m_, while YOLOV8-L demonstrates the best performance on mAP_l_. Furthermore, each model leveraged its respective pre-trained model during training to improve performance.

**Table 4.** Recall Comparison between Deformable DETR and our method

|  | **Deformable Detr Recall** | **Our Method Recall** |
| --- | --- | --- |
| van | 0.898 | **0.916** |
| adult | 0.766 | **0.779** |
| suv | 0.935 | **0.937** |
| car | 0.925 | **0.928** |
| bus | **0.988** | 0.981 |
| motorcycle | **0.722** | 0.719 |
| bicycle | 0.887 | **0.899** |
| child | 0.617 | **0.684** |
| microcar | 0.873 | **0.915** |
| Small truck | 0.949 | **0.983** |
| rickshaw | 0.842 | **0.895** |
| other | 0.778 | **0.955** |
| Motor tricycle | 0.927 | **0.935** |
| truck | 0.990 | 0.990 |
| Special vehicle | 1.000 | 1.000 |
| Other non-motor | **0.250** | 0.222 |
| Blurred vehicle | **0.571** | 0.500 |
| average recall | 0.819 | **0.837** |
